# Development of High Temperature Resistant Stereocomplex PLA for Injection Moulding

**DOI:** 10.3390/polym14030384

**Published:** 2022-01-19

**Authors:** Sebastian Körber, Kevin Moser, Jan Diemert

**Affiliations:** Department of Polymer Engineering, Fraunhofer Institute for Chemical Technology ICT, 76327 Pfinztal, Germany; kevin.moser@ict.fraunhofer.de (K.M.); jan.diemert@ict.fraunhofer.de (J.D.)

**Keywords:** poly(L-lactide), PLA, stereocomplex, nucleating agent, crystallisation, compounding, injection moulding

## Abstract

In this study, the production of stereocomplex PLA formulations (sc-PLA) by compounding and subsequent injection moulding at different mould temperatures was investigated. Several selective nucleating agents were identified and compounded with different poly(L-lactide)/poly(D-lactide) (PLLA/PDLA) ratios on a co-rotating twin screw extruder. The effect of nucleating agents (NA) on the crystallisation behaviour of the compound was systematically investigated by DSC analysis. The crystallisation behaviour of NA-21 (aluminium complex of a phosphoric ester), also in combination with talc, under cooling rates of up to 70 K/min was analysed. The wide-angle X-ray diffraction (WAXD) results showed a complete stereocomplex (sc) crystal formation on all specimens containing NA-21 even at the highest cooling rates. The thermo-mechanical testing of sc-PLA shows a Young’s modulus of approx. 3 GPa, yield stress of 30–40 MPa, elongation of 1%, and a heat deflection temperature (HDT-B) up to 180 °C. Processing sc-PLA exclusively via the processing route of compounding and injection moulding will open new areas of application for PLA at higher temperatures.

## 1. Introduction

The new millennium brought increasing awareness about the use of fossil resources influencing the climate to such an extent that human life could be plunged into a crisis. In this context, in 2018, the EU Commission presented its strategy for a competitive, climate-neutral economy by 2050 [1]. To achieve these goals, fossil resources must be phased out and replaced by renewable ones. Besides converting energy sources, a paradigm shift in materials production is required. In the field of plastics, which are conventionally produced from fossil resources, biopolymers with the potential of a significantly better CO_2_ balance can make a decisive contribution [2].

One of these biopolymers, which can be produced from starch and sugar, is poly-(lactic acid) (PLA). PLA has good mechanical properties compared to other biopolymers and is already used in commodity and biomedical applications [3].

Since the monomer of PLA (lactic acid) is enantiomeric, PLA can be divided into poly(L-lactide) (PLLA), poly(D-lactide) (PDLA), and poly (DL-lactide) (PDLLA). While PLLA and PDLA are semi-crystalline polymers with a melting point of approx. 180 °C, PDLLA is not crystallizable due to the atactic configuration of the polymer chain. However, due to the semirigid molecular backbone, the crystallisation rate of PLLA and PDLA is rather slow. This results in an amorphous product after injection moulding [4], as the modulus greatly drops above the glass transition temperature (T_g_ ≈ 55 °C) [5].

As mentioned above, PLA has favourable properties compared to other biopolymers but falls behind other petroleum-based polymers, inhibiting the wider use of PLA, especially in technical applications. Various modifications have been studied to improve the (thermo-)mechanical properties, e.g., blending with other polymers [6,7,8], usage of plasticizers [9,10], copolymerisation using other monomers [11,12,13], use of functional fillers [14,15,16] and nucleating agents [17]. However, for some methods, its biodegradability and recyclability often suffer [18,19].

Another promising method to improve the thermal properties of PLA is the formation of a stereocomplex (sc) between enantiomeric mixtures of PLLA and PDLA, which result in sc-PLA with an approx. 50 °C higher melting temperature due to the tight packing of the enantiomeric chains through the H-bond interaction [20,21,22]. PLA can form sc crystals in the melt, in the solid stage, and in the solution [23,24]. The resulting sc-PLA has many unique properties such as high mechanical strength, modulus, and toughness [25,26], good thermo-mechanical properties [27], good solvent resistance, and low thermal and hydrolytic degradation rates [28,29]. In summary, these properties make sc-PLA an interesting candidate as a high-performance biopolymer.

The formation of the sc-crystals cannot be easily controlled because PLLA and PDLA have to be mixed on a molecular level and at the optimum ratio to form the sc crystal. The key factor in controlling the formation of the sc is the molecular weight of the polymer chains (MW). For low-molecular-weight (LMW) PLAs, the sc crystallisation is favourable, but for high molecular weights (HMW), the sc crystallisation is significantly suppressed and the individual crystallisation of PLLA and PDLA is predominant [23,24].

LWM PLAs are not suitable for technical applications as they do not have the necessary mechanical properties and processability. For this reason, the formation of fully sc crystalline PLA must be achieved for HMWs to produce heat-resistant PLA for technical applications.

The literature describes many methods for improving the sc-crystallisation for HWMs, such as stereoblock copolymerization [30,31], supercritical fluid treatment [32], control over macromolecular topologies [33,34], melt blending at low temperature [35], thermal treatments by annealing at high temperature [26], stretching-induced phase transition [36,37], and the use of nucleating agents [38].

However, for use in industrial applications and the implementation of sc-PLA, the processing and shaping must be feasible in easy, cheap, scalable, and rapid processing procedures, like extrusion and injection moulding. Here the approach of controlling the sc crystallisation by a suitable NA is feasible. Ideally, these NAs promote sc crystallisation and suppress homopolymeric crystallisation. In this context, L. Han et al. coined the term “selective nucleator” [39]. This term is used in subsequent publications and can also be attributed to some of the previously published studies by Urayama, [38,40,41,42]. However, most of these studies are based on solution casting or processing on lab scale equipment and thus are not validated for up-scaling in larger quantities.

In the present study, feasible selective nucleators were determined by screening experiments on a laboratory scale twin-screw extruder. Based on these selected nucleation agents were used to compound a pilot scale co-rotating twin screw extruder. The compounds were injection moulded into tensile bars, and thermo-mechanical properties were investigated.

## 2. Materials and Methods

### 2.1. Materials

PLLA L130 (Mw = 168 kg/mol, polydispersity index Mw/Mn = 2.02, GPC analysis based on PMMA standard) and PDLA D070 (Mw = 87 kg/mol, polydispersity index Mw/Mn = 1.73, GPC analysis based on PMMA standard) were purchased from Total Corbion (Gorinchem, The Netherlands). All polymers have an optical purity of at least 99%. Finntalc-M03 was used as a reference nucleator and was purchased from Mondo Minerals B.V. (Amsterdam, The Netherlands). Following additives were examined: NA-21 (provided by ADEKA Europe, Düsseldorf, Germany), TMB-5 (provided by Shanxi Provincial Institute of Chemical Industry, Taiyuan, China), PPZn (synthesised according to the description in [43]) and the plasticizer PEG 400 and PEG 1500 (purchased from Sigma-Aldrich, Darmstadt, Germany) were examined. Table 1 shows the naming of the samples produced in this paper, the corresponding publications, and the preparation method used in these publications. The mass fractions of the respective NAs were based on the corresponding publications.

### 2.2. Sample Preparation

**Figure 1:**
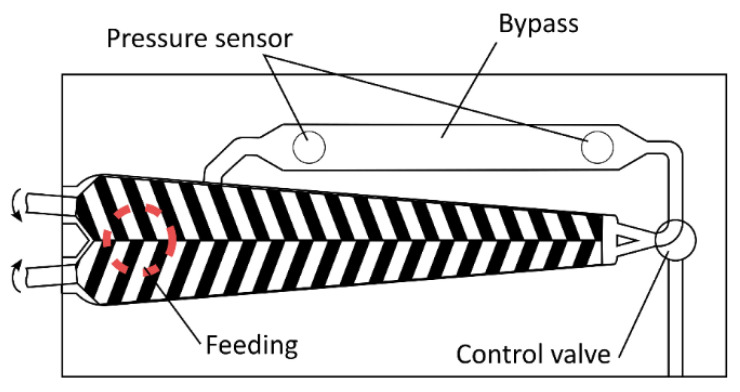
Haake MiniLab.

For the laboratory experiments, PLLA and PDLA granulates were milled and mixed with the nucleator. The mixture was then dried in a vacuum oven at 80 °C for 8 h. Compounding was carried out on a Haake MiniLab at a temperature of 220 °C (see Figure 1). The extruder has a bypass channel that allows the material to circulate and a specific residence time to be set before the valve is opened and samples can be taken. The residence time was set to 1.5 min to match the approx. residence time of the pilot scale extruder and therefore achieve comparable thermal degradation.

**Figure 2:**
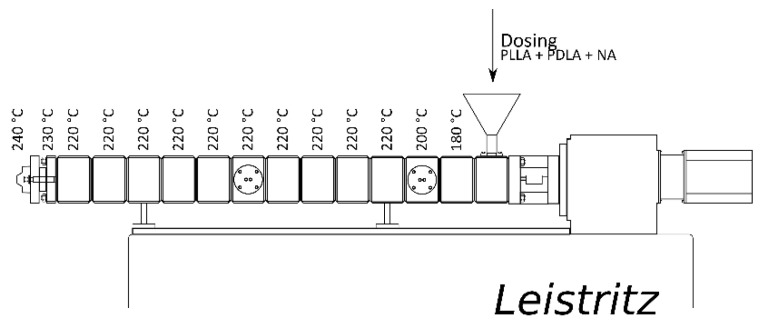
Experimental setup pilot scale extrusion.

For the experiments carried out in the pilot scale, the PLA granulates were dried for 8 h at 80 °C in a Heliomat pre-drying station. Compounding was carried out on a co-rotating twin screw extruder type Leistritz ZSE 27 HP. The experimental setup and temperature profiles are shown in Figure 2. Injection moulding of the tensile bars was done on an Engel ES200 at different mould temperatures to investigate the influence on the crystallinity of the samples.

### 2.3. Measurements

#### 2.3.1. Differential Scanning Calorimetry (DSC)

Thermal analysis of the samples was carried out using DSC (DSC 1, Mettler Toledo, Columbus, OH, USA). Samples of about 10 mg were weighed and placed into open aluminium pots. For non-isothermal measurements, the samples were heated from 20 to 250 °C to erase the thermal history. Afterward, the samples were cooled down again to 20 °C. To analyse their characteristic in the second heating step, the samples were again heated up to 250 °C. Cooling and heating rates were varied through the experiments and are given in the respective section. The crystallisation temperature (T_c_) and enthalpy of crystallisation (Δh_c_) were obtained from the cooling sequence, while the cold crystallisation peak temperature (T_cc_), enthalpy of cold crystallisation (Δh_cc_), melting point temperature (T_m_), and melting enthalpy (Δh_m_) were recorded from the 2nd heating step. The following equation was used to calculate the degree of crystallinity within the samples:

$$X_{c}=\frac{\Delta h_{m}}{\Delta {h_{m}}^{0}}\times 100\%$$

Δh_m_ is the measured endothermic enthalpy of melting. The theoretical melting enthalpy of 100% crystalline PLA was taken to be $\Delta h_{m,\mathrm{sc}}^{0}=144\;J/g\;$ for the sc crystals and $\Delta h_{m,\mathrm{hm}}^{0}=93\;J/g$ for the HM-crystals [46].

#### 2.3.2. Wide-Angle X-ray Diffraction (WAXD)

WAXD experiments were performed on a Bruker D8 Advance at room temperature using CU radiation (wavelength of 1.54 Å). The scanning rate was 0.05°/s in the scattering angle range of 2θ = 10 − 25°. The degree of crystallinity was determined with DIFFRAC.EVA V5.1 from Bruker AXS (Billerica, MA, USA).

#### 2.3.3. (Thermo-)Mechanical Test

Dog bone samples according to Typ 1A DIN EN ISO 3167 were injection moulded at different mould temperatures. Before every thermo-mechanical analysis, the specimens were stored for at least 24 h under standard climate conditions (23 °C, 50% humidity). Tensile properties were measured according to DIN EN ISO 527-1 on a universal testing machine Inspekt Table 10 kN from Hegewald & Peschke (Nossen, Germany) at a crosshead speed of 50 mm/min.

The heat deflection temperature test machine HDT 3 VICAT from Cheast (High Wycombe, UK) was used to measure the HDT values according to ISO 75-1. The test specimens were tested at a temperature gradient of 120 K/h and a starting temperature of 26 °C. The bending stress was 0.45 MPa and thus corresponds to method HDT-B.

## 3. Results

### 3.1. Screening Nucleating Agents

**Figure 3:**
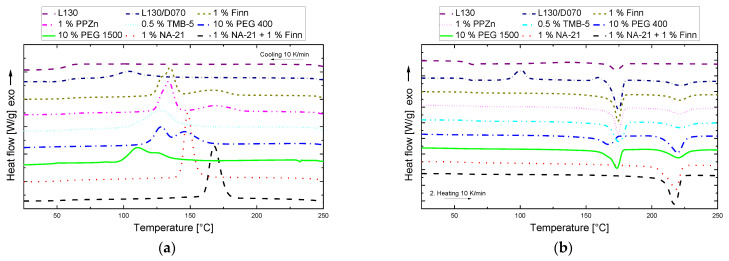
DSC analysis, screening NAs (**a**) cooling (250 to 20 °C) rate 10 K/min, (**b**) heating (20 to 250 °C) rate 10 K/min.

First, screening with the available NAs was carried out. The aim was to select the best NAs in order to reduce the number of pilot scale experiments. To reveal the most suitable NAs for manufacturing sc-PLA, DSC measurements were performed after compounding on the MiniLab. Figure 3a,b show the cooling and second heating sequence of all investigated compounds of L130 and D070 (L130/D070) with a specific amount of NA (see Table 2 for corresponding data). The pure PLA L130 solidifies without any crystallisation peaks due to the slow crystallisation rate of PLA. However, for the pristine PLA L130/D070, a crystallisation peak can be observed at about 110 °C. This can be explained by the following reasons. Firstly, L130/D070 contains PLA with shorter chain lengths, and the crystallisation speed generally doubles with a halving of the chain length [47]. Secondly, sc crystallisation is much faster than the crystallisation of homo crystals (hm) [48]. Additionally the sc-crystals can act as NAs for hm crystallisation [49,50,51,52]. Compared to L130/D070, crystallisation with 1% Finn begins at 185 °C and has a bimodal form. The crystallisation occurring at higher temperatures can be assigned to sc crystallisation, the lower to homo (hm) crystallisation. The following curve with 1% PPZn, shows practically identical cooling behaviour as the Finn sample. The TMB-5 sample has a very similar crystallisation behaviour to the non-nucleated sample L130/D070, but due to the heterogeneous nucleation, crystallisation can be observed at higher temperatures. The short-chain PEG 400 shows a crystallisation behaviour with two clearly recognizable peaks, while the higher molecular PEG 1500 shows only an elongated crystallisation curve with a barely recognizable bimodality. The remaining two samples with NA-21 both show a distinct sharp crystallisation peak at about 150 and 170 °C, respectively.

For PLLA/PDLA systems, a cautious interpretation of the DSC heating curves is necessary because sc crystals can crystalize during the melting of α crystals [37]. Therefore, DSC heating curves can only be used to determine pure sc crystalline or mixed α and stereo crystals. WAXD measurements which do not change the state of the sample were performed for absolute determination at a later stage for the pilot scale samples.

In the curve of L130 and L130/D070, cold crystallisation is noticeable. The L130/D070 sample shows a small exothermic peak at about 160 °C, which can be traced back to melt recrystallisation, which can either be the transformation of imperfect hm crystals in the neighbourhood of sc crystals or the recrystallisation of imperfect α’ to α crystals [50,53].

All curves with NAs show absence of cold crystallisation, indicating that the samples solidified thermodynamically stable. The specimens with the nucleator Finn, PPZn, TMB-5, PEG 400, and PEG 1500 show a very similar melting behaviour with two endothermic melting peaks at approx. 175 °C and 220 °C, which can be attributed to the melting of the hm crystals and the sc crystals, respectively. While the melting points of the samples considered so far hardly changed, compounding with 10% PEG leads to a decrease of the hm melting point to 166 °C. The remaining two NAs, NA-21 and NA-21 + Finn, finally show the intended heating behaviour with a single melting peak (Δhm,sc ≈ 23–37 J/g) at about 217 °C, which indicates the formation of pure sc crystals.

Only two of the investigated selective NAs allowed the achievement of pure sc crystallisation. In the following, possible reasons for the failure of the other NAs are discussed. The authors using PPZn [39] as selective NA attribute its effect to the good lattice matching between sc crystals and the NA (approximately equal b-axis of the crystal lattices). PLLA and PDLA chains both interact well with the NA and bind epitaxially to the nucleus surface. This increases the probability that PLLA and PDLA chains will form complex structures and, at the same time, prevents intramolecular folding of the PLLA or PDLA chains, which would lead to hm crystallisation. Since the recorded cooling curves show no similarity to the publication even to higher or lower concentrations [39], it is reasonable to conclude that this mechanism was ineffective in our experiments. The great similarity to the cooling curve of the talc compound described above suggests that PPZn was only effective as a simple heterogeneous nucleus in the melt and could not specifically promote stereo crystal formation.

TMB-5 is an aryl amide derivative that tends to form three-dimensional dendritic networks in polymer melts [54]. For this purpose, TMB-5 must first dissolve in the PLA mixture and then forms fine networks via hydrogen bonds and self-organisation when cooled down [55]. With a suitable surface, the PLLA and PDLA chains can thus attach themselves to the formed needle-like superstructure. This leads to targeted nucleation of the sc crystals [40]. The formation of the TMB-5 network, which is important in this case, is strongly dependent on temperature and time. The chosen experimental parameters not being suitable to generate a sufficient network for sc formation, cannot be excluded in this study. Therefore, sc crystallisation was only slightly promoted. The strong temperature and time dependence make the NA TMB-5 impractical for later processing in injection moulding.

The increase in enthalpy of the sc crystals by PEG cannot be explained by heterogeneous nucleation since the melting point of the two PEG variants is 5 °C (PEG 400) and 50 °C (PEG 1500), respectively. Thus, the two materials are liquid in the crystallisation temperature range of the PLA. In this case, PEG acts much more as a plasticiser, which leads to an increase in chain mobility. As a result, the energy required for folding the chains decreases and the crystallisation rate increases. Thus, more sc crystals can be formed [41]. The T_g_ is a very good indication of this increase in mobility. Unfortunately, this could only be determined for the sample PEG 1500 (T_g_,_PEG 1500_ = 46 °C) at the selected temperature gradients. Since the miscibility increases with decreasing chain length of PEG and T_g_ decreases further, the T_g_ of the PEG 400 sample should be even lower than that of the PEG 1500 sample [56].

This could explain the higher enthalpy of sc crystals of the PEG 400 sample compared to the PEG 1500 after the 2nd DSC heating step sample. However, in the publication [41], lower T_g_ was measured despite longer chain lengths of the PLA. This suggests that higher chain mobilities could be achieved, which may have led to a higher stereo complexity.

Understanding the sc nucleation of NA-21 is more complex. The authors of the corresponding publication [38] attribute the nucleation of the aluminum complex to the intrinsic delay of the PLA chain motion after sc crystallisation. This is caused by the sc crystallisation being faster and taking place at higher temperatures. Consequently, the sc crystalline regions are no longer available for hm crystallisation, which is consequently hindered. The presence of NA-21 is expected to intensify this effect by, i.e., completely suppressing hm crystallisation and thus leading to exclusive sc crystallisation. It can be stated that NA-21 promotes sc crystallisation very well, but is also a good NA for hm crystals in the absence of the respective enantiomeric chain [38]. Therefore, it can be concluded that NA-21 can interact with the homopolymeric chains itself, as well as with the blends of PLLA and PDLA. Suppression of the hm crystallisation or ensuring a completed sc crystallisation by its nucleation at the comparatively high crystallisation temperatures of 150–170 °C is possible. Which process takes place cannot be concluded with enough certainty from the data. However, it can be stated that NA-21 has many polar structures and end groups which enables the possibility to promote epitaxial attachment of the L- and D-chains and thus create pure stereo complexity.

### 3.2. Influence of the Cooling Rate on the Crystallisation Behaviour

**Figure 4:**
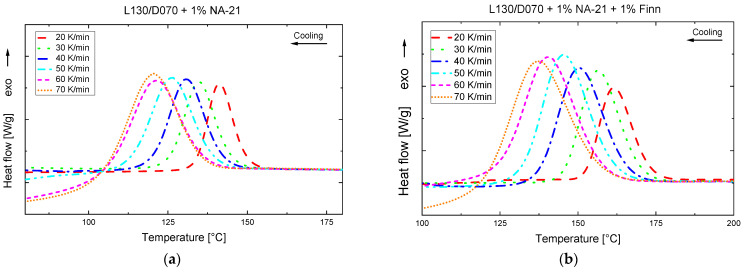
DSC-Analysis, variation of the cooling rate 20–70 K/min (250 to 20 °C) (**a**) L130/D070 + 1% NA-21 (**b**) L130/D070 + 1% NA-21 + 1% Finn.

The previous studies have shown that pure sc-PLA nucleation was achieved for the NA-21 and NA-21 + Finn samples at cooling rates of 10 K/min. Since injection moulding has significantly higher cooling rates, another DSC analysis study with cooling rates from 20 to 70 K/min was performed. The cooling rate of 70 K/min corresponded to the maximum cooling rate of the analytic device. The corresponding DSC cooling curves are shown in Figure 4. As the cooling rate increases, the curves shift to lower temperatures, which can be explained by the influence of the cooling rate on the nucleation process. The driving force for crystallisation is the undercooling of the melt, which influences the required thickness of the critical nucleus. The size of the critical nucleus is inversely proportional to the undercooling. At high temperatures (low undercooling), thicker critical nuclei are produced, paired with a long incubation time (time required at a certain temperature to form a nucleus above the critical radius [57]). If the temperature drops, the undercooling increases and the size required to form critical nuclei decreases [58]. The lower the cooling rate, the higher the residence time in the respective temperature interval. Nucleation does not occur until the residence time and the incubation time are equalized. At low cooling rates, the equalization process already takes place at higher temperatures, whereas at high cooling rates there is insufficient time for stable nucleation and the crystallisation is shifted to lower temperatures with increasing cooling rate.

**Figure 5:**
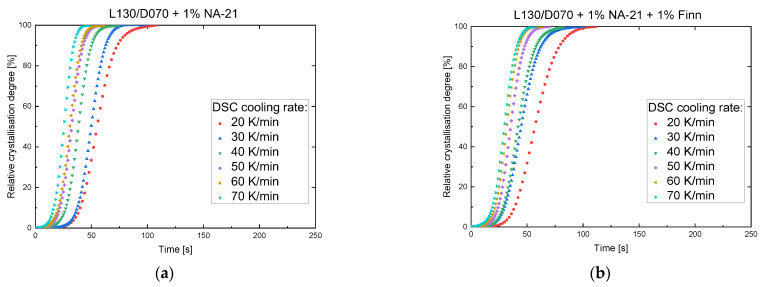
Relative degree of crystallisation depending on the crystallisation time for different cooling rates (**a**) L130/D070 + 1% NA-21 (**b**) L130/D070 + 1% NA-21 + 1% Finn.

When examining the relative degree of crystallisation (see Figure 5), it can be observed that crystallisation is completed faster due to the higher cooling rate. By lowering the crystallisation temperature, the energy barrier for nucleation decreases, as already described above, which increases the nucleation rate. This allows the process of crystallisation to be completed faster. Looking at the crystallisation enthalpies of NA-21 and Finn (see Table 3), the melt enthalpy decreases continuously with higher cooling rates. This results from the temperature interval and time during crystallisation shifting to lower temperatures and shorter times. Therefore, the chain mobility, important for the crystal growth, becomes lower, and thus fewer areas can crystallize.

**Figure 6:**
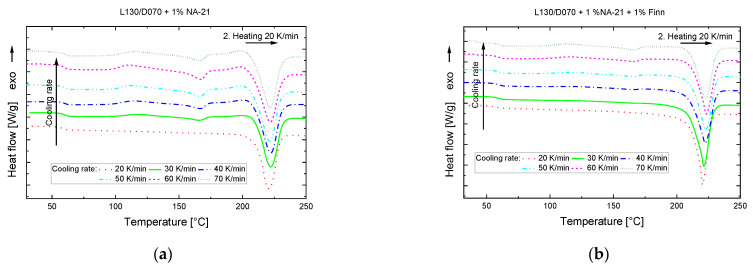
DSC analysis, 2. Heating 20 to 250 °C, 20 K/min after variation of the cooling rate 20–70 K/min (**a**) L130/D070 + 1% NA-21 (**b**) L130/D070 + 1% NA-21 + 1% Finn.

The PLA sample with 1% NA-21 no longer shows pure sc melt peaks in the 2nd heating step at a cooling rate of 30 K/min (c.f. Figure 6). From this temperature gradient onwards, NA-21 no longer promotes sc crystal formation to such an extent that only sc crystals are formed. In the heating curve, there are melting peaks of the hm crystals at approx. 165 °C in addition to the sc melting peaks at 220 °C. Together with the cold crystallisation and the melt crystallisation in which hm crystals are converted into sc crystals, the sc melting enthalpy of NA-21 in Table 3 could be falsified. When looking at the 2nd heating curve of the PLA sample NA-21 + Finn, no melt or cold crystallisation peaks are observed, which would indicate hm crystals or a thermodynamically unstable solidification.

The investigations of the cooling rate clearly show that the cooling rate has a much greater influence on the nucleating effect of the NA-21 than on NA-21 + Finn. Accordingly, high mould temperatures must be selected for the injection moulding tests in order to guide the cooling process in such a way that the material has sufficient time for a predominant sc crystallisation.

### 3.3. Injection Moulding

L130/D070 in combination with Finn, NA-21, and NA-21 + Finn was compounded in pilot scale and afterwards injection moulded into tensile bars. Finn was used as a reference for the selective NAs. Injection moulding parameters are shown in Table A1 in the Appendix A. Mainly the mould temperature was varied;; other parameters were only adjusted to counteract injection moulding errors. The specimens containing 1% Finn could only be injection moulded up to a mould temperature of 121 °C, as they did not have sufficient strength for demoulding at higher mould temperatures under acceptable cooling times. The opposite happened for samples containing NA-21. Even at the high mould temperatures, these were already sufficiently stable for demoulding at short cooling times. However, their high brittleness proved to be problematic for these samples, so some samples were destroyed when the ejector pins moved out during the demoulding step.

**Figure 7:**
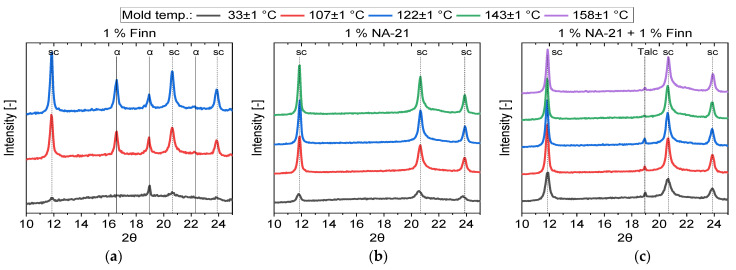
WAXD diffractogram after injection moulding of L130/D070 with different NAs at different mould temperatures (**a**) 1% Finn (**b**) 1% NA-21 (**c**) 1% NA-21 + 1% Finn.

After injection moulding, the specimens were examined for their crystal structure using WAXD (Figure 7). Due to the different unit cells of the α and sc crystals, different diffraction patterns were obtained, which allow for a differentiation between α and sc crystals. The diffraction angles 14.3–15°, 16–17°, 18.5–19° and 22.5–23.5° [59,60] can be assigned to the α crystals, the diffraction angles 11.5–12°, 20.5–21° and 23.5–24° [61,62] to the sc crystals.

As assumed, the sample containing 1% Finn has both α and sc crystals, with the sc peaks being slightly more prominent than those of the α crystals. The two samples with NA-21 are both purely sc crystallized for all mould temperatures. The small peak at 19° in the samples with NA-21 + Finn can be attributed to the diffraction of talc [63].

Evaluations of crystallinity (Table 4) show that crystallinity tends to increase with rising mould temperature. The Finn sample solidifies practically amorphously in the cold mould and reaches moderate crystallinities with increasing mould temperatures. In comparison, the samples nucleated with NA-21 are semi-crystalline even at cold mould temperatures and reach high crystallinities of above 40% with increasing mould temperatures. It must be noted that the crystallinity between XRD and DSC cannot be compared since the evaluation using XRD is strongly dependent on the angular range considered and the approximation of the amorphous range.

In the DSC investigations (see Section 3.2), it was found that pure sc crystallisation with NA-21 is no longer possible for temperature gradients equal to or higher than 30 K/min. However, the WAXD results show that hm crystallisation no longer occurs even when the material is injected into the cold mould (fast cooling rates) with NA-21. For this effect, several reasons are possible. On the one hand, processing in injection moulding adds a process step, which leads to further thermal degradation and thus to shorter chains, which promotes sc-crystallisation. On the other hand, the macromolecules are aligned by the shear deformation of the melt during the injection process. This alignment could simplify the arrangement of PLLA and PDLA chains and thus additionally support sc crystallisation.

The WAXD results show that sc-PLA could be exclusively produced in the compounding and injection moulding process. 

### 3.4. (Thermo-) Mechanical Measurements

**Figure 8:**
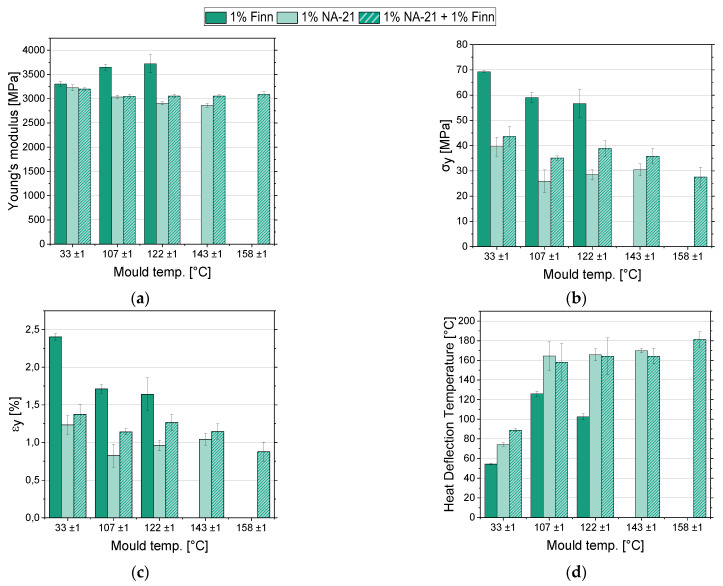
(Thermo-) mechanical analysis, variation of the mould temperatures, L130/D070 in combination with 1% Finn, 1% NA-21, 1% NA-21 + 1% Finn, (**a**) Young’s modulus, (**b**) yield stress, (**c**) elongation, (**d**) HDT-B.

To determine the (thermo-)mechanical properties, the injection moulding specimens were examined using tensile and HDT testing. The results of the examination are summarized in Figure 8.

Figure 8a shows the distribution of the Young’s modulus of the three NA combinations. For the pure sc crystallized samples NA-21 and NA-21 + Finn, the Young’s modulus, starting from about 3.2 GPa, decreased with the change from cold to hot mould and then remained relatively stable. In the samples nucleated with Finn, the Young’s modulus rose continuously to approx. 3.7 GPa as the mould temperature increased.

Figure 8b and c show the yield stress and yield strain of the three samples. It can be observed that in all three variants, the yield stress decreased with the switch to the hot mould. In contrast, for higher mould temperatures it remained relatively constant, with the NA-21 + Finn samples showing a slight decrease and the NA-21 samples a slightly increasing tendency of yield stress. The samples nucleated with Finn show higher strengths than the other two samples with yield stresses of 55–70 MPa. Similar behaviour can be observed for the characteristic values of the yield strain. When switching to the hot mould, the yield strain decreased and remained relatively constant, considering the standard deviations.

There are only a few publications addressing the mechanical properties of sc-PLA. For example, Tsuji and Ikada investigated the mechanical properties of solvent-cast polymer films that are difficult to compare with the results of this study due to the different processing routes (Young’s modulus 0.8–1.7 GPa, elongation at yield 3–4%, and yield stress of approx. 10–45 MPa [depending on the chain length]) [25]. A comparison with oriented sc fibers showed that the tensile strength was about 20 times higher and the elongation at break almost 30 times higher (σ_B_ = 90 kg/mm^2^, e_B_ = 30%) for the solution spinning process [49]. For the economically more beneficial melt spinning process, tensile strength was approx. 10 times higher (σ_max_ = 48 cN/tex) [64]. For neat L130, the datasheet shows a Young’s modulus of 3.5 GPa, tensile strength = 50 MPa, elongation at break ≤ 5% [44]. The pure sc samples thus have a slightly lower Young’s modulus at approx. 3 GPa, while the samples with α and sc crystals show a slightly higher Young’s modulus at 3.7 GPa.

The elastic behaviour of semi-crystalline thermoplastics is essentially determined by the intramolecular forces between the molecular chains. Since the intermolecular bonds in crystalline areas of semi-crystalline thermoplastics are higher than in amorphous areas, the modulus of elasticity generally increases with increasing crystallinity [65]. This is also the case for PLA, although a non-linear increase in the modulus of elasticity with crystallinity can be observed due to the influence of the α and α’ crystals [66]. Since the modulus of elasticity is almost proportional to the melting temperature (also depending on the intermolecular forces) [65], the Young’s modulus of sc-PLA should be higher than that of hm crystals. This behaviour could not be observed with the specimen containing NA-21. The yield stress, elongation at yield, and impact strength are also determined by the crystallinity. Thus, the densely packed crystallites ensure the hardness and strength of the polymer, and the amorphous areas are responsible for a certain elasticity and impact strength of the polymer [67]. As a result, the yield stress generally increases with crystallinity and the elongation at yield and impact strength decrease [65]. The elongation at yield corresponds to the behaviour expected from the literature and decreases with increasing crystallinity. The yield stress, however, shows a contrary behaviour, as it decreases with increasing crystallinity. As already described, the yield strain and impact strength are more dependent on the amorphous areas than on the crystalline areas of the polymer. As these two parameters show the expected behaviour and the yield stress, which depends more on the crystalline areas, shows unexpected behaviour, it can be concluded that the decrease of the yield stress is caused by the crystalline areas with increasing mould temperature or crystallinity. One possible reason for this contradictory observation is the changing crystal morphology. With increasing mould temperature, the cooling gradient in the sample decreases. Consequently, the temperature of the melt approaches the crystallisation temperature more slowly. This leads to a lower nucleating frequency and faster crystal growth rate due to higher chain mobility [59,68,69]. Therefore, we assume that with an increasing mould temperature, the nucleation frequency decreases and the crystal growth rate increases, resulting in a coarser spherulitic structure. Compared to the fine crystalline structure, this results in comparable poor properties and tends to be more prone to brittle fracture.

Figure 8d shows the result of the HDT tests. The HDT-B values in the cold mould are comparably low (approx. 55–90 °C), but increase significantly for all material systems with NA-21 as NA and at higher mould temperatures (HDT-B ≈ 160–180 °C). In the cold mould, the NA combination of NA-21 + Finn shows the highest heat resistance. However, when the mould temperature is increased to 107-143 °C, NA-21 shows slightly better heat resistance than the NA combination NA-21 + Finn. With the highest selected mould temperature (158 ± 1 °C), injection moulding with NA-21 was no longer possible. The highest measured HDT-B of all samples with an HDT value of 181 °C was achieved with NA-21 + Finn.

The sample nucleated with Finn shows the worst heat resistance in the cold mould. This is explained by practically amorphous solidification of the sample (Table 4). When the T_g_ (≈57 °C) is reached, the chains change from the energy-elastic state into the entropy elastic or viscous state. Chain sliding is easier to achieve and thus leads to a fast failure of the sample. The other two samples did not solidify amorphously despite the low mould temperatures. Accordingly, the crystalline structures is able to prevent the chains from slipping when the T_g_ is exceeded, thus increasing the HDT-B. As the mould temperature rises and the crystallinity grows, the HDT-B increases significantly. It can be observed, however, that the mixed α and sc crystalline samples have a significantly lower HDT-B than the pure sc crystalline samples. This may be due to the lower crystallinity, as well as to the sc-crystals, which are tougher at high temperatures. Between the mould temperatures of 107–143 °C, the HDT-B of the purely sc crystallising samples changes only slightly, which is probably related to mere marginal changes in crystallinity of only a few percent. Due to the slightly higher crystallinity, the NA-21 samples show a slightly higher HDT-B. However, a comparison at a mould temperature of, e.g., 120 °C shows that crystallinity cannot be the only factor. At this temperature, the HDT-B only differs by about 3 °C, but the crystallinity by almost 10%. This could indicate that the NA-21 + Finn sample has a more homogenous, more heat-resistant crystal morphology. It could also explain why the NA-21 + Finn sample reaches the highest HDT-B at a mould temperature of 158 °C, despite showing lower crystallinities than the NA-21 samples.

A comparison with the datasheet of L130 shows (HDT-B amorphous 60 °C, crystalline 105 °C, mould temperature 90–100 °C, 3–7% NA (D070 was used as NA) [44]) that the HDT-B of the amorphous samples is in similar ranges. With the mixed α- and sc-crystalline samples, an increase in HDT-B to over 120 °C is possible. With the pure sc samples, however, it is possible to increase the HDT-B temperature by approx. 80 °C (compared to hm PLA). 

## 4. Conclusions

Several NAs known from literature were compounded on a laboratory scale extruder. Crystallisation behaviour was studied via DSC analysis. NA-21 and NA-21, in combination with Finn, induced the sole crystallisation of the stereocomplex crystal, while all other tested NAs could not achieve a pure sc crystallisation. DSC investigations with cooling rates up to 70 K/min revealed that NA-21 has a greater dependency on the cooling rate than NA-21 + Finn. The compounding and injection moulding process was transferred into the pilot scale. During injection moulding, the mould temperature was varied in order to influence the crystallinity. Exclusive sc crystallinity in all specimens with NA-21 was verified via WAXD. Mechanical testing showed a Young’s modulus of approx. 3 GPa, σ_y_ of 30–40 MPa and ε_y_ of approx. 1% for all sc samples. (Thermo-)Mechanical measurements revealed HDT-B temperatures of approx. 160 °C for the samples processed with mould temperatures of 107–143 °C. With the highest tested mould temperature of 158 °C, the highest HDT-B of more than 180 °C could be achieved. This proves that an increase of the heat resistance of PLA via sc crystallisation of approx. 80 °C (compared to PLA [44]) is possible. However, elongation and yield strength drop slightly compared to neat PLA. Further development is needed to preserve or even increase the ductility while maintaining a high level of heat resistance. In addition, process parameters should be studied systematically to gain more process stability and have better insights into their influence on the mechanical properties of sc-PLA. In summary, this study has shown that it is possible to produce sc-PLA via compounding and injection moulding with suitable NAs. This could enable the cost-effective use of sc-PLA in a wide range of more demanding applications.

## Figures and Tables

**Table 1:** Naming the examined specimen.

Name	Matrix	Additive	Publication	Processing Method
L130	L130	-	[44]	-
L130/D070	L130 + D070	-	-	-
Finn	L130 + D070	1% Finntalc-M03	-	-
NA-21	L130 + D070	1% NA-21	[38,45]	Solvent casting, chloroform
NA-21/Finn	L130 + D070	1% NA-21 + 1% Finntalc-M03	[38,45]	Solvent casting, chloroform
TMB-5	L130 + D070	0.5% TMB-5	[40]	Solvent casting, chloroform
PPZn	L130 + D070	1% PPZn	[39]	Solvent casting, chloroform
PEG 400	L130 + D070	10% PEG 400	[41]	Solvent casting, dichloromethane
PEG 1500	L130 + D070	10% PEG 1500	[41]	Solvent casting, dichloromethane

**Table 2:** Thermal properties of PLLA/PDLA blends with different NAs obtained in non-isothermal DSC analysis after melt crystallisation (cooling rate = 10 K/min) and subsequent heating (heating rate = 10 K/min).

Name	T_c_ (C)	Δh_c_ (J/g)	T_c, sc_ (°C)	Δh_c, sc_ (J/g)	T_cc_ (°C)	Δh_cc_ (J/g)	T_m, hm_ (°C)	Δh_m, hm_ (J/g)	T_m, sc_ (°C)	Δh_m, sc_ (J/g)
L130	-	-	-	-	136.2	12.2	172.6	12.8		
L130/D070	104.1	14.1	-	-	101.1	17.5	174.2	40.5	220.8	13.8
1% PPZn	134.2	35.8	168.9	15.7	-	-	173.9	39.8	220.8	15.3
1% Finntalc-M03	135.3	38.2	165.8	15.4	-	-	173.9	40.7	220.6	14.2
0.5% TMB-5	128.1	47.1	-	-	-	-	174.0	41.3	221.1	15.6
10% PEG 400	128.3	24.2	146.1	25.1	-	-	166.4	24.1	219.0	37.5
10% PEG 1500	110.6	24.9	126.3	13.7	-	-	172.9	35.0	219.6	23.7
1% NA-21	-	-	149.0	54.3	-	-	-	-	217.5	52.2
1% NA-21 + 1% Finn	-	-	168.4	59.3	-	-	-	-	216.7	57.2

**Table 3:** Thermal properties of PLLA L130/PDLA D070 blends with NA-21 and NA-21 + Finn obtained in non-isothermal DSC analysis after melt crystallisation (cooling rates = 20–70 K/min) and subsequent heating (heating rate = 20 K/min).

	Rate (K/min)	T_c_ (°C)	Δh_c_ (J/g)	Δh_cc_ (J/g)	T_m, hm_ (°C)	Δh_m, hm_ (J/g)	T_m, sc_ (°C)	Δh_m, sc_ (J/g)
1% NA-21	20	142.1	40.9	-	-	-	219.7	45.5
	30	135.9	35.3	3.1	165.4	3.7	221.1	46.1
	40	131.5	31.7	4.6	165.8	5.7	220.7	47.3
	50	127.6	27.9	6.0	166.1	6.1	220.4	47.8
	60	123.0	28.9	6.7	166.2	7.1	220.7	50.8
	70	121.4	27.2	6.0	165.5	5.6	220.8	51.8
1% NA-21 + 1% Finn	20	162.6	56.0	-	-	-	219.0	54.6
	30	157.2	51.5	-	-	-	220.6	55.5
	40	151.4	47.8	-	-	-	221.9	51.4
	50	147.5	44.4	-	-	-	221.4	48.4
	60	142.5	41.1	-	-	-	221.6	48.5
	70	137.6	37.6	-	-	-	222.0	45.7

**Table 4:** Crystallinities calculated from WAXD diffractograms.

Name	Mould Temp. (°C)	X_c,sc_ (%)	X_c,hm_ (%)
1% Finn	33 °C	3.44	1.23
	107 °C	23.68	7.08
	123 °C	25.90	7.63
1% NA-21	33 °C	19.17	-
	106 °C	39.47	-
	121 °C	46.10	-
	142 °C	48.05	-
1% NA-21 + 1% Finn	33 °C	30.62	-
	108 °C	39.45	-
	123 °C	37.59	-
	143 °C	41.36	-
	158 °C	43.41	-

## Appendix A

**Table A1:** Injection moulding parameters.

Name	Mould Temp. (°C)	Injection Pressure (bar)	Injection Time (s)	Holding Pressure (bar)	Holding Time (s)	Cycle Time (s)
Finn	33	200	2.51	30	10	46
	106	200	2.05	35	50	126
	121	200	2.05	35	50	126
NA-21	33	200	2.51	40	10	50
	106	200	2.09	50	10	47
	121	200	2.29	10	10	47
	142	200	2.05	10	10	47
NA/Finn	33	200	0.5	45	3	19
	106	200	2.05	10	10	47
	121	200	2.05	10	10	47
	142	200	2.05	35	15	53
	158	200	2.05	35	15	53
